# A 20-year retrospective cohort study of TB infection among the Hill-tribe HIV/AIDS populations, Thailand

**DOI:** 10.1186/s12879-016-1407-4

**Published:** 2016-02-09

**Authors:** Tawatchai Apidechkul

**Affiliations:** School of Health Science, Mae Fah Luang University, Chiang Rai, 57100 Thailand

**Keywords:** HIV/AIDS, TB, Hill-tribe, ARV, Thailand

## Abstract

**Background:**

A retrospective cohort study was conducted to determine the situation, trend, and factors associated with TB infection, and factors related to the life status among the HIV/AIDS Hill-tribe in Northern Thailand. Hill-tribe people have been migrating to and formed settlements along the Thai border areas for many decades. There are now having 1.6 million people of 6 different groups–Akha, Lahu, Lisu, Hmong, Yao and Keren–each with a specific culture, language and lifestyle. The Hill-tribe becomes a new vulnerable of HIV and TB infections in Thailand.

**Methods:**

A systematic data-reviewing approach was used to identify the information from the rosters of ARV clinics, OPD cards, and laboratory reports from 16 hospitals in Chiang Rai Province, Thailand. The data were collected from the first reported HIV/AIDS case of the Hill-tribe to the end of 2010. A chi-square test and logistic regression models were used to identify associations at the significance level of alpha = 0.05.

**Results:**

A total of 3,130 cases were included in the study. The majority of patients were Akha (46.0 %) followed by Lahu (19.7 %), 54.6 % were males, 44.6 % were 26–35 years old. The major risk factor of HIV infection was sexual intercourse (93.1 %); 23.9 % were still alive at the date of data collection, 30.7 % were diagnosed with pulmonary TB. The Akha Hill-tribe HIV/AIDS individuals had a greater chance of TB infection than did Yao individuals with OR_adj_ = 1.50 (95 % CI = 1.01-1.92). Females had a greater chance of TB infection than males with OR_adj_ = 1.33 (95 % CI = 1.11-1.59); being classified as HIV and AIDS groups had a greater chance of TB infection than those asymptomatic group with OR_adj_ = 11.59 (95 % CI = 7.19-18.71), and OR_adj_ = 1.71 (95 % CI = 1.03-2.87); and not having received the ARV group had a greater chance of TB infection than those who having received the ARV group with OR_adj_ = 2.59 (95 % CI = 2.09-3.22). The patients who had been diagnosed with HIV infection during 1990–1995 and 1996–2000 had less chance of TB infection than those who were diagnosed from 2006–2010, with OR_adj_ = 0.04 (95 % CI = 0.01-0.14) and 0.11 (95 % CI = 0.07-0.17), respectively. Regarding life status; females had a better chance of being still alive at the date of data collection than being males with OR_adj_ = 1.41 (95 % CI = 1.19-1.66). Those who had a defined route of transmission in the category of “mother-to-child” and “IDU” had a better chance of being still alive compared to those who contracted HIV from “sexual intercourse,” with OR_adj_ = 2.05 (95 % CI = 1.56-2.18), and OR_adj_ = 8.45 (95 % CI = 1.55-46.13), respectively.

**Conclusions:**

Thailand needs to create a TB and HIV/AIDS surveillance system for Hill-tribe populations to determine the situation and trend and to develop an appropriate model for providing care at the earlier stage of HIV/AIDS infection to prevent later TB infection.

## Background

The Millennium Development Goals (MDGs) has set eight goals for the most pressing development challenges, with a set of measurable time-bound targets. The sixth goal is “…. having halted by 2015 and begun to reverse the incidence of major serious infectious diseases such as the Human Immunodeficiency Virus (HIV) Infection, and Tuberculosis (TB).” Tuberculosis control targets are to halt and begin to reverse the rising incidence of TB and to halve the 1990 prevalence and death rates by 2015, and 22 million lives worldwide have been saved from tuberculosis since 1995 [1].

HIV/AIDS is still a major global health problem, having claimed more than 39 million lives since the first HIV case report; the great majority of sufferers live in developing countries, particularly Africa and Asia. Globally, by the end of 2013, 1.5 million people had died from HIV-related causes, and approximately 35.0 million people were living with HIV, with 2.1 million being newly detected cases [2].

Tuberculosis is a communicable bacterial infection that is second only to HIV/AIDS as the greatest killer worldwide due to a single infectious agent. TB is also the most significant opportunistic infection among AIDS patients, causing one fourth of all HIV-related deaths worldwide. In 2013, nine million people had tuberculosis, of which 1.5 million died, representing a decrease in TB mortality of 45.0 % between 1990 and 2013 [3] due to the effort of innumerable health-related agencies striving to reach the MDG target of an 85.0 % reduction of TB incidence worldwide. In fact, the goal had been exceeded for the fourth consecutive year [1]. Still, more than 95.0 % of tuberculosis deaths occur in low-and middle-income countries.

The population of Thailand reached 67,222,972 in October 2014 [4]. Due to the development of economics, medical sciences, and public health services, Thai life expectancy has increased to 73.6 years on average, 71.3 years for males and 76.1 years for females [4]. However, it is widely accepted that AIDS has reached an epidemic proportion in Thailand particularly in northern and middle regions. Approximately 2.0 % of males and 1.5 % of females live with HIV/AIDS, while the figure was 0.8 % globally in 2011 [5]. Since the first AIDS case was reported in Thailand in 1984, there have been 388,621 cumulative cases of HIV/AIDS, and 100,617 have died [6]. Tuberculosis is the highest occurring (29.5 %) opportunistic infection among HIV/AIDS in Thailand [6].

In 2013, there were an estimated 1.1 million TB-HIV co-infections globally, 78.0 % in sub-Saharan Africa. TB is the leading cause of death among people living with HIV, accounting for some 360,000 people who die of HIV-associated TB annually, with no difference between males and females [7]. Approximately one-third of people living with HIV/AIDS worldwide have latent tuberculosis, and these individuals are at a 29 times greater risk of developing active TB disease than those without HIV. These individuals are also at a greater risk of contracting drug-resistant TB, of which a delay in diagnosis increases mortality [7].

Thailand is one of the 22 countries with a high burden of both TB as identified by the World Health Organization [8]. In 2011, the HIV/AIDS co-infection prevalence in Thailand was 161/100,00 [8]. Risky behaviors and insufficient knowledge about HIV remain at alarmingly high levels among the sexually active age group in marginalized populations, such as the Hill-tribe people in Thailand.

**Fig. 1:**
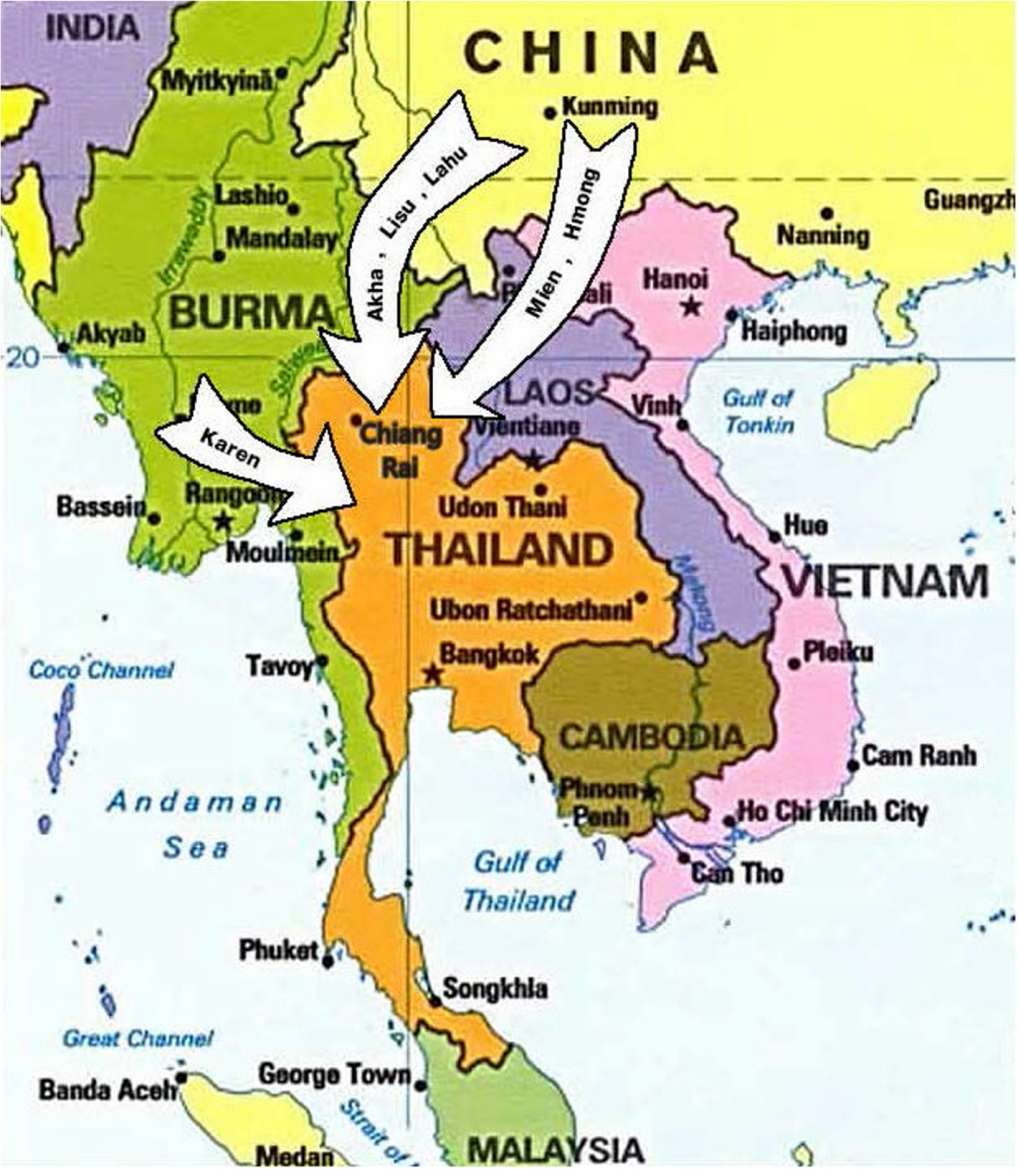
Hill-tribe migratory map in Thailand. Mien and Hmong had the same migratory route from the south of China through the Republic of Laos to northern Thailand. Akha, Lahu and Lisu had the same migratory route from the south of China through the Republic of Laos to Northern Thailand. Karen had migrated into western region of Thailand. The figure is adapted from http://www.hilltribe.org/thai/museum/. The figure’s holder is The Mirror Foundation, Thailand, and has been premised to use by the director of The Mirror Foundation, Thailand

**Fig. 2:**
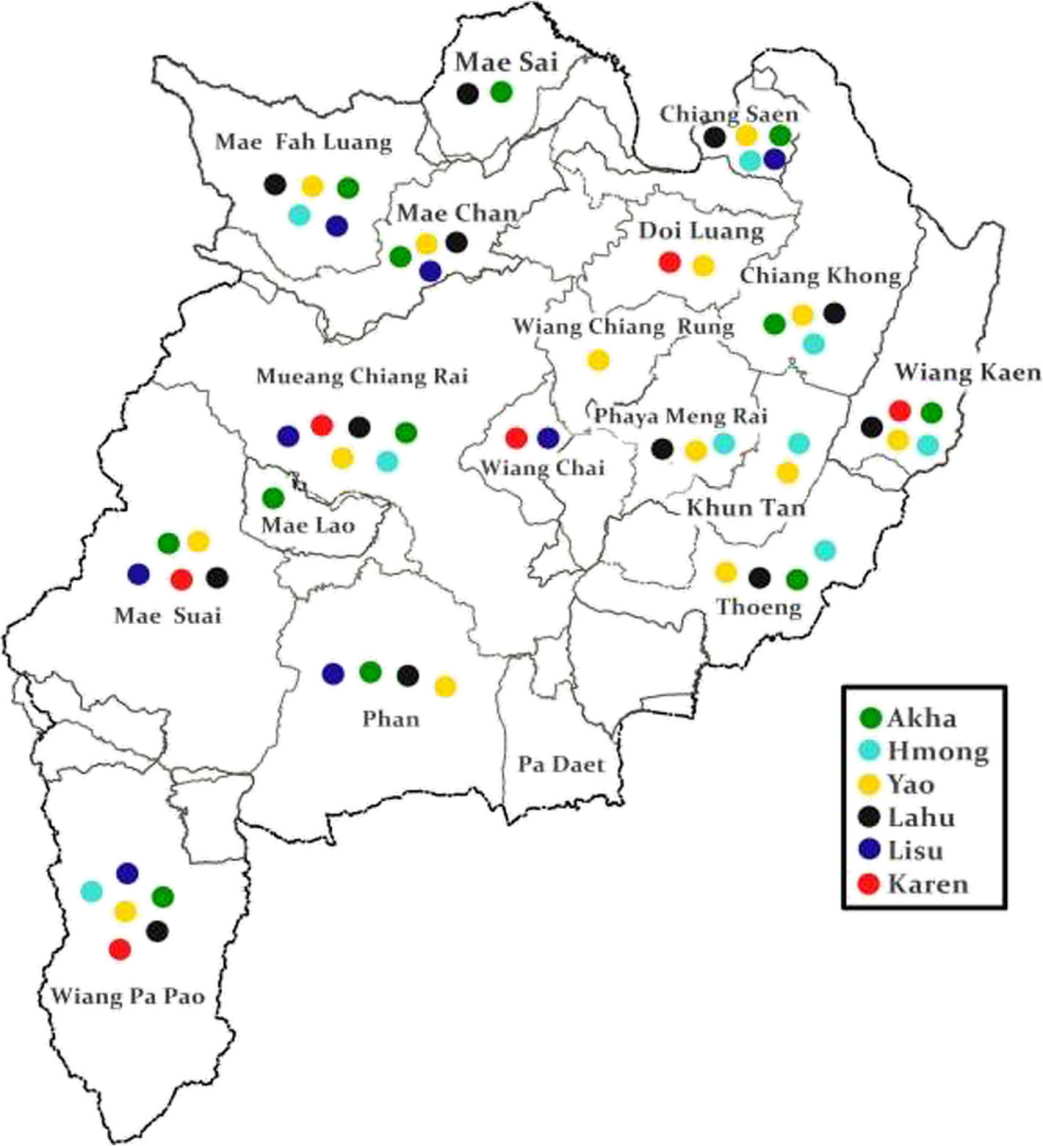
Different destination sites of the Hill-tribe in Chiang Rai Province, Thailand. Most of Akha, Yao, and Lahu have settled throughout Chiang Rai Province. Hmong has settled in eastern of Chiang Rai Province. Karen and Lisu have settled in the western of Chiang Rai Province

Thailand has computerized population database as all Thais are required by law to register for an Identification (ID) card by age 7. A Thai ID card with ID number is essential for most legal or formal transactions in Thailand including claiming access to government free or subsidized health service. Hill-tribe people have been migrating to and formed settlements along the Thai border areas for many decades (Fig. 1). They are now living in different locations in Chiang Rai Province, Thailand (Fig. 2). Their settlements are gradually becoming more permanent with later generations but there is still a tradition of Hill-tribe people crisscrossing the border according to their economic, cultural or political necessity from time to time. Their villages are often in very remote areas making applying for a house registration or address either difficult or unnecessary. Their status is rather like one of an alien refugee even well into a second or third generation of immigrants. Successive Thai governments, being concerned about border security, have taken a cautious approach toward conferring Thai citizenship to Hill-tribe people by granting different types and stages of internal passport and citizenship. Many still have not obtained documentation which entitles them to travel legally within an allowed area due to lack of information, language barrier, fear of arrest etc., which considerably restricts their movement including seeking employment, education for children, and medical treatment. Also traditional belief and medical system is still strong in their villages and which many may choose to manage their health and illnesses.

Hill-tribes in Thailand are classified into six main groups [9]: Lahu, Akha, Lisu, Karen, Yao and Kmong. These tribes have immigrated from the south of China to Thailand in the last several decades. Approximately 1,600,000 tribe people were living in Thailand in 2013 [9]. Each group has their own language, culture and beliefs, which are different from those of the Thai. Most live in the mountainous border areas in north of Thailand among 16 provinces. The Hill-tribe people of Chiang Rai Province, the northernmost province of Thailand, live in 652 villages with a total population of 180,214 persons [10]. Their villages are in remote areas approximately 70 km from the capital city with poor road access and public transport. Many Hill-tribe people do not qualify for or have failed to obtain a Thai identification card, which generally is required for free or subsidized government medical and educational services. Most of the Hill-tribe people, if working, work in manual jobs with low pay. The Hill-tribe people in northern Thailand therefore still have limited opportunity to earn money, receive health information, and have access to affordable healthcare. These populations are vulnerable to infectious diseases, such as HIV and TB.

There is no information regarding the TB and HIV/AIDS status among the Hill-tribes of Thailand. Through a systematic review of the secondary information from 16 hospitals in Chiang Rai Province, this study aimed to determine the situation, trend, and factors associated with TB among the Hill-tribe HIV/AIDS population who had received care from those hospitals during a twenty years period between 1990 and 2010.

## Methods

### Study design

A retrospective cohort study was conducted to investigate the situation, trend, and factors associated with TB infection among Hill-tribe HIV/AIDS patients who had visited one of the 16 hospitals in Chiang Rai Province, Thailand, from the first reported HIV case (who was a Hill-tribe patient) until 2010.

### Study population

The study population constituted Hill-tribe patients who were living in one of the 652 Hill-tribe villages in Chiang Rai Province. These villages were made up of 36 Karen (7,628 persons), 59 Kmong (31,522 persons), 63 Yao (13,400 persons), 243 Akha (68,897 persons), 216 Lahu (48,835 persons), and 35 Lisu (9,932 persons) villages, with in total 180,214 persons.

### Study sites and source of data

Patients’ information were collected from the records of ARV clinics of the 16 hospitals in Chiang Rai Province, which were Chiang Rai Central, Wiang Pa Pao, Mae Chan, Wiang Ken, Chiang Saen, Mae Suai, Mae Fah Laung, Phaya Mengrai, Doi Luang, Phan, Chiang Khong, Wiang Chai, Thoeng, Khun Tan, Mae Sai, and Mae Lao hospitals. The author was granted permission to assess hospital records for analysis from the hospital director, and was granted to access data in the step of subjects’ identification from the chief of the district government office.

In this study, four data sources were used to complete the information: a) a government database to identify the tribe of the subjects; b) ARV rosters to collect general information, including history of TB infection; c) laboratory reports to support laboratory information; and d) OPD card for a complete medical record, including history of treatments and OI infections.

### Eligible population

The eligible population of the study was Hill-tribe patients who were living in Chiang Rai and had a diagnosis of HIV/AIDS who had visited one of the 16 hospitals during the period from when the first Hill-tribe HIV/AIDS patient was reported to the hospital system in 2010.

### Inclusion criteria

The inclusion criteria included patients whose medical records indicated that they were a) identified as Hill-tribe persons, b) living in the study areas, and c) medically diagnosed as having HIV/AIDS with or with out tuberculosis.

### Sample size

All of the HIV/AIDS patients in the rosters of 16 ARV clinics from the first case of HIV/AIDS in the t-tribe through 2010 were included in the study. TB diagnosis was made by a combination of clinical, radiological and laboratory methods based on the date of diagnosis. HIV/AIDS classification was done based on at the date of diagnosis.

### Research instruments

To gather and integrate information from hospital OPD cards and ARV clinic records, we developed a data collection form with three parts: a) basic information part for patient characteristics, such as age, sex, tribe, marital status, weight, height, drug use history, etc.; b) laboratory information, such as the CD4 level, viral load, CBC, sputum test, etc.; and c) clinical information, such as history, symptoms, signs, treatment, and health status (alive or dead) at the last record entry. The form was validated by Content Validity Index (CVI) method before use. TB and HIV/AIDS diagnosis was used.

### Data-gathering procedures

The collecting information form was developed after the literature review. The form was validated by three external experts in the field of TB-HIV/AIDS; one worked at School of Health Science, and another two worked at the Ministry of Public Health, Thailand, and piloted in a study at Mae Chan Hospital. The systematic reviewing process was tested in Mae Chan Hospital before commencing the project.

Written permission for access information was granted by the director of each hospital and the chief of each ARV clinic. The information from each unit (ARV, OPD, and Laboratory) was linked by the hospital number. Before reviewing and investigating the information in any section of the hospital, the ARV staff helped to identify the cases that met the inclusion criteria.

In the verification process of the Hill-tribe people, we first used the database of the district government office system to identify the residential location of the patients, including village and patient’s names, and second, the information from the ARV was used to confirm verification process to ensure that all of the subjects that were included in the study met the inclusion and exclusion criteria.

### Statistical analysis

The data were double-entered by two different persons and validated using Microsoft Excel. A data analysis was carried out using STATA version 8.2 (Stata Corp, College Station, TX), and Epi-Info version 6.04d (US Centers for Disease Control and Prevention, Atlanta, GA).

Both descriptive and inferential statistics were used to analyze the information. For a descriptive analysis, the mean, standard deviation, minimum, and maximum were used to describe the characteristic of continuous variables, while the percentage or proportion was used to describe the characteristic of categorical variables.

A chi-square test and logistic regression were used to detect the association between independent and dependent variables at critical values of 0.10 in the univariate model and 0.05 in the multivariate model.

### Ethical consideration

This research study was approved by The Ethics in Human Research Committee of Mae Fah Luang University. Permission to collect data in nine hospitals in Chiang Rai Province was granted by The Chiang Rai Provincial Public Health Office. Patient records and information was anonymized and re-identified prior to analysis.

## Results

There were 3,130 Hill-tribe HIV/AIDS patients from 16 hospitals in Chaing Rai Province between 1990 and 2010 who were recuited for the study based on the criteria. Of these cases, 960 (30.7 %) had at least one episode of TB. The first case of HIV/AIDS in the Hill-tribe people was a 25-year-old Lisu, reported at Mae Suai Hospital, Chiang Rai Province, in 1990.

The majority of the tribes were Akha (46.0 %), followed by Lahu (19.7 %) and Yao (9.5 %). More than half were male (54.6 %), and 44.6 % were 26–35 years old, followed by 36–45 years old (25.2 %), and 16–25 years old (14.3 %). The peak period of HIV/AIDS infection among the Hill-tribes occurred from 2001–2005 (43.9 %), followed by 2006–2010 (33.7 %).

Regarding the habitat, 25.8 % of the people resided in the Mae Fah Laung district, followed by 18.8 % in the Mae Suai district, 16.2 % in the Muang Chiang Rai district, and 11.1 % in Mae Chan district. Regarding the occupations, 44.8 % were agricultural workers and 32.2 % were daily and temporary employees. The major risk factors of HIV infection were sexual intercourse (93.1 %), 6.4 % mother-to-child, and 0.5 % IDU. Most of the subjects did not recive ARV (66.7 %), only 297 had a measured CD4 level, 23.9 % were still alive at the date of data collection, and 30.7 % had TB disease documented at some time after the HIV/AIDS infection was diagnosed (Table 1).

**Table 1:** General characteristics of the hill tribe HIV/AIDS

Characteristics	Number	Percent
Total	3,130	100.0
Tribal group		
Akha	1,441	46.0
Karen	271	8.7
Kmong	221	7.1
Lahu	617	19.7
Lisu	282	9.0
Yao	298	9.5
Sex		
Male	1,710	54.6
Female	1,420	45.4
Age (years)		
<15	230	7.4
16-25	448	14.3
26-35	1,396	44.6
36-45	790	25.2
46-55	197	6.3
>56	69	2.2
Max = 90, Min = 0, Mean = 31.83		
Occupation		
Agriculture	1,403	44.8
Temporary employee	1,007	32.2
Trader	57	1.8
Housewife	98	3.1
Children (<7 years)	109	3.5
Students (7 > university)	104	3.3
Unemployed	89	2.8
Others	263	8.4
Year of diagnosis		
1990-1995	154	4.9
1996-2000	548	17.5
2001-2005	1,374	43.9
2006-2010	1,054	33.7
Living areas (District)		
Muang Chiang Rai	507	16.2
Wiang Pa Pao	442	14.1
Mae Chan	348	11.1
Wiang Ken	123	3.9
Chiang Saen	41	1.3
Mae Suai	589	18.8
Mae Fah Laung	808	25.8
Phaya Menrai	28	0.9
Doi Luang	40	1.3
Phan	73	2.3
Chiang Khong	58	1.9
Wiang Chai	18	0.6
Thoeng	14	0.5
Khun Tan	2	0.1
Mae Sai	14	0.5
Mae Lao	25	0.8
Diagnosis as (classified in medical record)		
Asymptomatic	373	11.9
HIV (AIDS related complex)	2,060	65.8
AIDS	697	22.3
Route of transmission		
Sexual intercourse	2,913	93.1
Mother to child	200	6.4
IDU	17	0.5
Life status (last entry)		
Alive	749	23.9
Dead	2,381	76.1
Initiated ARV		
Yes	1,042	33.3
No	2,088	66.7
CD4 level (cell/ml.)		
>200	285	9.1
<200	12	0.4
Unknown	2,833	90.5
Max = 281, Min = 32, Mean = 117.74		
TB disease		
No	2,170	69.3
Yes	960	30.7

Table 2 shows the proportion of TB-positive and-negative cases in various factors; eight variables had a statistically significant difference -- tribal groups, risk factors, survival status, sex, age, occupation, diagnosis, and receiving ARV at a significance level of 0.50. Regarding the tribes, 33.7 % of Akha-HIV and 32.6 % of Lahu-HIV had at least one episode of TB, which was greater than the other tribes. Female-HIV group had a greater proportion of TB disease (34.8 %) than the male-HIV group (27.3 %). HIV/AIDS patients who worked as trader and temporary employees groups had a 43.9 % and 41.0 % incidence of TB disease.

**Table 2:** Comparison between TB-HIV and non TB-HIV patients

Characteristics	TB		χ^2^-test	*p*-value
	No (%)	Yes (%)		
Tribal group				
Akha	955 (66.3)	486 (33.7)	15.780	0.008*
Karen	200 (73.8)	71 (26.2)		
Kmong	157 (71.0)	64 (29.0)		
Lahu	447 (72.4)	170 (27.6)		
Lisu	190 (67.4)	92 (32.6)		
Yao	221 (74.2)	77 (25.8)		
Route of transmission				
Sexual intercourse	1,975 (68.9)	893 (31.1)	7.727	0.026*
Mother to child	185 (75.8)	59 (24.2)		
IDU	9 (52.9)	8 (47.1)		
Survival status				
Alive	859 (82.4)	184 (17.6)	124.888	<0.001*
Dead	1,311(62.8)	776 (37.2)		
Sex				
Male	1,244 (72.7)	466 (27.3)	20.727	<0.001*
Female	926 (65.2)	494 (34.8)		
Age (year)				
<15	184 (80.0)	46 (20.0)	51.263	<0.001*
16-25	345 (77.0)	103 (23.0)		
26-35	955 (68.4)	441 (31.6)		
36-45	494 (62.5)	296 (37.5)		
46-55	133 (67.5)	64 (32.5)		
>56	59 (85.5)	10 (14.5)		
Occupation				
Agriculture	1,067(76.1)	336 (23.9)	106.869	<0.001*
Temporary employee	594 (59.7)	413 (41.0)		
Trader	32 (56.1)	25 (43.9)		
Housewife	79 (80.6)	19 (19.4)		
Children (<7 years)	93 (85.3)	16 (14.7)		
Students (7 > University)	73 (70.2)	31 (29.8)		
Unemployed	62 (69.7)	27 (30.3)		
Others	170 (64.6)	94 (35.4)		
Diagnosis				
Asymptomatic	353 (94.6)	20 (5.4)	298.643	<0.001*
HIV	1,220 (59.2)	840 (40.8)		
AIDS	597 (85.7)	100 (14.3)		
ARV				
Yes	1,312 (62.8)	776 (37.2)	124.384	<0.001
No	858 (82.3)	184 (17.7)		

*Significance level at *p*-value < 0.05

Table 3 shows the eight variables that were statistically significantly related to TB infection among the Hill-tribe HIV/AIDS patients in the simple logistic regression model at a significance level of 0.10. The Lisu and Akha groups had a greater chance of TB infection -- 1.40 and 1.46 times greater than that of the Yao, respectively. Being female was a factor associated with TB infection (1.42 times compared to being male), and the Hill-tribe HIV/AIDS patients age 26–35 and 46–55 had 1.85 and 1.92 times greater chance, respectively, of TB infection than did those aged less than 15 years.

**Table 3:** Simple logistic regression of the factors associated with of TB disease among the hill tribe HIV/AIDS

Factors	TB		OR (90 % CI)	p-value
	No (%)	Yes (%)		
Tribal group				
Akha	955 (66.3)	486 (33.7)	1.46 (1.15-1.85)	0.008*
Karen	200 (73.8)	71 (26.2)	1.02 (0.74-1.39)	0.922
Kmong	157 (71.0)	64 (29.0)	1.17 (0.84-1.62)	0.430
Lahu	447 (72.4)	170 (27.6)	1.10 (0.84-1.42)	0.584
Lisu	190 (67.4)	92 (32.6)	1.40 (1.03-1.87)	0.050*
Yao	221 (74.2)	77 (25.8)	1.00	
Sex				
Male	1,244 (72.7)	466 (27.3)	1.00	
Female	926 (65.2)	494 (34.8)	1.42 (1.25-1.62)	<0.001*
Age (year)				
<15	184 (80.0)	46 (20.0)	1.00	
16-25	345 (77.0)	103 (23.0)	1.19 (0.86-1.66)	0.374
26-35	955 (68.4)	441 (31.6)	1.85 (1.39-2.46)	<0.001*
36-45	494 (62.5)	296 (37.5)	2.40 (1.78-3.23)	<0.001*
46-55	133 (67.5)	64 (32.5)	1.92 (1.33-2.78)	0.004*
>56	59 (85.5)	10 (14.5)	0.67 (0.36-1.26)	0.306
Year of diagnosis				
1990-1995	151 (98.1)	3 (1.9)	0.035 (0.01-0.09)	<0.001*
1996-2000	524 (95.6)	24 (4.4)	0.081 (0.06-0.12)	0.041*
2001-2005	823 (59.9)	551 (40.1)	1.18 (1.03-1.35)	0.053*
2006-2010	672 (63.8)	382 (36.2)	1.00	
Occupation				
Agriculture	1,067 (76.1)	336 (23.9)	1.00	
Temporary employee	594 (59.0)	413 (41.0)	2.21 (1.91-2.56)	<0.001*
Trader	32 (56.1)	25 (43.9)	2.48 (1.58-3.89)	0.001*
Housewife	79 (80.6)	19 (19.4)	0.76 (0.49-1.17)	0.306
Children	93 (85.3)	16 (14.7)	0.55 (0.35-0.86)	0.030*
Student	73 (70.2)	31 (29.8)	1.35 (0.93-1.95)	0.181
Unemployed	62 (69.7)	27 (30.3)	1.38 (0.93-2.05)	0.175
Others	170 (64.4)	94 (35.4)	1.74 (1.37-2.19)	<0.001*
Diagnosis				
Asymptomatic	353 (94.6)	20 (5.4)	1.00	
HIV	1,220 (85.7)	840 (40.8)	12.15 (8.27-17. 86)	<0.001*
AIDS	597 (85.7)	100 (14.3)	2.95 (1.95-4.49)	<0.001*
Route of transmission				
Sexual intercourse	1,976 (68.9)	893 (31.1)	1.00	
Mother to Child	185 (75.8)	59 (24.2)	0.57 (0.42-0.76)	0.002*
IDU	9 (52.9)	8 (47.1)	1.96 (0.88-4.36)	0.170
ARV				
No	858 (82.3)	184 (17.7)	2.76 (2.37-3.31)	<0.001*
Yes	1,312 (62.8)	776 (37.2)	1.00	

*Significance level at *p*-value < 0.10

Mother-to-child transmission of HIV/AIDS patients was less likely to result in TB infection among the HIV/AIDS Hill-tribe patients compared to sexual intercourse, with OR = 0.57 (90 % CI = 0.42-0.76). Moreover, those who did not receive ARV presented as a greater chance of TB infection among the HIV/AIDS Hill-tribe, with OR = 2.76 (90 % CI = 2.38-3.31).

In the multiple logistic regression model, five variables had a statistically significant association with TB infection among the Hill tribe HIV/AIDS individuals. The Akha Hill-tribe HIV/AIDS individuals had a greater chance for TB infection than did the Yao, with OR_adj_ = 1.50 (95 % CI = 1.01-1.92). Females had a greater chance of TB infection than did males, with OR_adj_ = 1.33 (95%CI = 1.11-1.59). The HIV and AIDS groups had a greater chance of TB infection among the HIV/AIDS Hill-tribe people, with OR_adj_ = 11.59 (95 % CI = 7.19-18.71) and OR_adj_ = 1.71 (95%CI = 1.03-2.87) compared to those in the asymptomatic group. For the Hill-tribe HIV/AIDS individuals, not receiving ARV was a factor associated with TB infection, with OR_adj_ = 2.59 (95 % CI = 2.09-3.22). Patients who had been diagnosed with HIV infection from 1990–1995 and 1996–2000 were less likely to have TB compared to those who were diagnosed from 1990–1995, with OR_adj_ = 0.04 (95 % CI = 0.01-0.14) and OR_adj_ = 0.11 (95 % CI = 0.07-0.17), respectively (Table 4).

**Table 4:** Multiple logistic regression to identify the factors associated with TB disease among the hill tribe HIV/AIDS

Factors	TB		OR_adj_ (95 % CI)	*p*-value
	Yes (%)	No (%)		
Trible group				
Akha	955 (66.3)	486 (33.7)	1.50 (1.01-1.92)	0.041*
Karen	200 (73.8)	71 (26.2)	1.11 (0.72-1.71)	0.635
Kmong	157 (71.0)	64 (29.0)	1.59 (1.01-2.53)	0.055
Lahu	447 (72.4)	170 (27.6)	0.91 (0.64-1.30)	0.619
Lisu	190 (67.4)	92 (32.6)	1.17 (0.78-1.76)	0.439
Yao	221 (74.2)	77 (25.8)	1.00	
Sex				
Male	1,244 (72.7)	466 (27.3)	1.00	
Female	926 (65.2)	494 (34.8)	1.33 (1.11-1.59)	0.002*
Diagnosis				
Asymptomatic	353 (94.6)	20 (5.4)	1.00	
HIV	1,220 (59.2)	840 (40.8)	11.59 (7.19-18.71)	<0.001*
AIDS	597 (85.7)	100 (14.3)	1.71 (1.03-2.87)	0.040*
ARV				
No	585 (82.3)	184 (17.7)	2.59 (2.09-3.22)	<0.001*
Yes	1,312 (62.8)	776 (37.2)	1.00	
Year of diagnosis				
1990 - 1995	151 (98.1)	3 (1.9)	0.04 (0.01-0.14)	<0.001*
1996 - 2000	524 (95.6)	24 (4.4)	0.11 (0.07-0.17)	<0.001*
2001 - 2005	823 (59.9)	511 (40.1)	1.39 (1.15-1.68)	0.001*
2006 - 2010	672 (63.8)	382 (36.2)	1.00	

*Significance level at *p*-value < 0.05

Table 5 shows the nine variables in the simple logistic regression analysis that had a statistically significant association with the patients’ life status at the date of data collection (alive or dead from the medical record) of the Hill-tribe HIV/AIDS individuals. The Karen and Kmong had less chance of being still alive at the date of data collection compared to the Yao tribe, with OR = 0.58 (90 % CI = 0.43-0.77) and OR = 0.55 (90 % CI = 0.41-0.74), respectively. Patients aged 16 years and above had less chance of being still alive compared to those aged less than 15 years, with OR = 0.51 (90 % CI = 0.37-0.69), OR = 0.60 (90 % CI = 0.46-0.79), OR = 0.60 (90 % CI = 0.45-0.80), and OR = 0.33 (90 % CI = 0.20-0.53) respectively. Those who had been diagnosed with HIV and AIDS had less chance of being still alive compare to those asymptomatic group, with OR = 0.06 (95 % CI = 0.04-0.12), and OR = 0.25 (90 % CI = 0.10-0.47) respectively. Those who had been infected through mother-to-child transmission and IDU had a better chance of being still alive at the date of data collection, with OR = 2.05 (90 % CI = 1.56-2.68) and OR = 8.45 (90 % CI = 1.55-46.13), respectively. Patients who did not receive ARV had less chance of being still alive at the date of data collection than those who received ARV, with OR = 0.15 ( 90 % CI = 0.12-0.19), and those who had an occupation in the categories of “temporary employee,” “housewife,” “students”, and “others” had a better chance of being still alive at the date of data collection compared to “agriculture,” with OR = 1.88 (90 % CI = 1.66-2.19), OR = 1.61 (90 % CI = 1.10-2.36), OR = 5.46 (90 % CI = 3.21-9.30), and OR = 1.74 (90 % CI = 1.36-2.23), respectively. Patients who had been diagnosed from 1990–1995, 1996–2000, and 2001–2005 had less chance of being still alive at the date of data collection compared with those who had been diagnosed between 2006 and 2010, with OR = 0.03 (90 % CI = 0.01-0.08), OR = 0.06 (90 % CI = 0.04-0.09), and OR = 0.82 (90%CI = 0.71-0.95) respectively.

**Table 5:** Simple logistic regression analysis of factors related to patients’ life status among the hill tribe HIV/AIDS

Factors	Life status		OR (90 % CI)	*p*-value
	Alive (%)	Dead (%)		
Tribal				
Akha	343 (23.8)	1,098 (76.2)	1.02 (0.82-1.28)	0.1 36
Karen	65 (24.0)	206 (76.0)	0.58 (0.43-0.77)	0.048*
Kmong	59 (26.7)	162 (73.3)	0.55 (0.41-0.74)	0.045*
Lahu	151 (24.5)	466 (75.5)	1.05 (0.82-1.35)	0.116
Lisu	72 (25.5)	210 (74.5)	1.31 (0.97-1.77)	0.100
Yao	59 (19.8)	239 (80.2)	1.00	
Sex				
Male	355 (20.8)	1,355 (79.2)	1.00	
Female	394 (27.7)	1,026 (72.3)	1.47 (1.28-1.68)	<0.001*
Age (year)				
<15	39 (17.0)	191 (83.0)	1.00	
16-25	69 (15.4)	379 (84.6)	0.51 (0.37-0.69)	0.024*
26-35	341 (24.4)	1,055 (75.6)	0.60 (0.46-0.79)	0.014*
36-45	248 (31.4)	542 (68.6)	0.60 (0.45-0.80)	<0.001*
46-55	50 (25.4)	147 (74.6)	0.87 (0.60-1.27)	0.074
>56	2 (2.9)	67 (97.1)	0.33 (0.20-0.53)	0.009*
Diagnosis				
Asymptomatic	11 (2.9)	362 (97.1)	1.00	
HIV	662 (32.1)	1,398 (67.9)	0.06 (0.04-0.12)	<0.001*
AIDS	76 (10.9)	621 (89.1)	0.25 (0.10-0.47)	<0.001*
TB				
No	142 (6.5)	2028 (93.5)	1.00	
Yes	607 (63.2)	353 (36.8)	24.56 (20.51-29.41)	<0.001*
Route of transmission				
Sexual intercourse	710 (24.4)	2,203 (75.6)	1.00	
Mother to child	31 (15.5)	169 (84.5)	2.05 (1.56-2.68)	0.038*
IDU	8 (47.1)	9 (52.9)	8.45 (1.55-46.13)	0.003*
ARV				
No	676 (32.4)	1412 (67.6)	0.15 (0.12-0.19)	<0.001*
Yes	73 (7.0)	969 (93.0)	1.00	
Occupation				
Agriculture	282 (20.1)	1,121 (79.9)	1.00	
Temporary employee	314 (31.2)	693 (68.8)	1.88 (1.66-2.19)	<0.001*
Trader	17 (29.8)	40 (70.2)	0.95 (0.60-1.50)	0.078
Housewife	15 (15.3)	83 (84.7)	1.61 (1.10-2.36)	0.051*
Children (<7 years)	10 (9.2)	99 (90.8)	1.03 (0.73-1.44)	0.067
Students (7 > University)	31 (29.8)	73 (70.2)	5.46 (3.21-9.30)	0.020*
Unemployed	25 (28.1)	64 (71.9)	0.31 (0.21-0.45)	0.053
Others	5 (20.9)	208 (79.1)	1.74 (1.36-2.23)	0.047*
Year of diagnosis				
1990-1995	2 (1.3)	152 (98.7)	0.03 (0.01-0.08)	<0.001*
1996-2000	15 (2.7)	533 (97.3)	0.06 (0.04-0.09)	<0.001*
2001-2005	389 (28.3)	985 (71.7	0.82 (0.71-0.95)	0.024*
2006-2010	343 (32.5)	711 (67.5)	1.00	

*Significance level *p*-value < 0.10

From the multiple logistic regression model at a significance level of 0.05, five variables were statistically significantly associated with patients’ life status at the date of data collection. The Karen and Kmong had less chance of being still alive compared to the Yao, with OR_adj_ = 0.58 (95 % CI = 0.43-0.77), and OR_adj_ = 0.55 (95 % CI = 0.41-0.74), respectively. Females had a better chance of being still alive than males, with OR_adj_ = 1.41 (95 % CI = 1.19-1.66). Patients who were diagnosed with “HIV” and “AIDS” had less chance of being still alive than those who were diagnosed with “Asymptomatic,” with OR_adj_ = 0.05 (95 % CI = 0.03-0.09), and OR_adj_ = 0.34 (95 % CI = 0.17-0.64), respectively. Those who had a defined route of transmission in the category of “mother-to-child” and “IDU” had a better chance of being still alive compared to those who contracted HIV from “sexual intercourse,” with OR_adj_ = 2.05 (95 % CI = 1.56-2.68) and OR_adj_ = 8.45 (95 % CI = 1.55-46.13), respectively. Finally, patients who did not receive ARV had less chance of being still alive compared to those who received ARV, with OR_adj_ = 0.11 (95 % CI = 0.08-0.14) (Table 6).

**Table 6:** Multivariate analysis of factors related to patients’ life status among the hill tribe HIV/AIDS

Factors	Life status		OR_adj_ (95 % CI)	*p*-value
	Alive (%)	Dead (%)		
Tribal group				
Akha	456 (31.6)	985 (68.4)	0.85 (0.82-1.28)	0.483
Karen	122 (45.0)	149 (55.0)	0.58 (0.43-0.77)	0.025*
Kmong	102 (46.2)	119 (53.8)	0.55 (0.41-0.74)	0.019*
Lahu	192 (31.1)	425 (68.9)	1.05 (0.82-1.35)	0.375
Lisu	75 (26.6)	207 (73.4)	1.30 (0.97-1.77)	0.485
Yao	96 (32.2)	202 (67.8)	1.00	
Sex				
Male	664 (37.7)	1,060 (62.3)	1.00	
Female	399 (28.1)	1,021 (71.9)	1.41(1.19-1.66)	<0.001*
Diagnosis				
Asymptomatic	144 (38.6)	229 (61.4)	1.00	
HIV	729 (38.4)	1,268 (61.6)	0.05 (0.03-0.09)	<0.001*
AIDS	107 (15.4)	590 (84.6)	0.34 (0.17-0.64)	0.001*
Route of transmission				
Sexual intercourse	992 (34.6)	1,877 (65.4)	1.00	
Mother to child	50 (20.5)	194 (79.5)	2.05 (1.56-2.68)	<0.001*
IDU	1 (5.9)	16 (94.1)	8.45 (1.55-46.13)	0.020*
ARV				
No	676 (32.4)	1412 (67.6)	0.11 (0.08-0.14)	<0.001*
Yes	73 (7.0)	969 (93.0)	1.00	

*Significance level *p*-value < 0.05

## Discussion

Our study illustrates that among 3,130 members of the Hill-tribe HIV/AIDS population, major factors associated with TB infection were being from the Akha tribe, being female, HIV and AIDS stages, and non-receiving ARV compared to the others tribes, being male, asymptomatic stage, and receiving ARV, respectively. Regarding factors related to patients’ survival status, we found that being of the Karen and Kmong tribes, non-receiving ARV, and HIV and AIDS stages had less of an opportunity to survive compare to those of being from other tribes, receiving ARV, and asymptomatic stage, respectively.

This study has a major limitation in the quality of the available secondary data. The identification and confirmation of a patient’s Hill-tribe status was a significant challenge. Fortunately, in identifying which patient on the hospital lists was a Hill-tribe person, we could rely on the characteristics of their first and last names, which provide a reliable clue to whether they met the inclusion criteria. The naming tradition is that the child takes the father’s last name (which is recorded on the hospital registration) even if he/she has grown up and chosen later to belong to a different tribe than that of his/her parents. The local ARV clinic staff had been extremely helpful in this respect, as most of them have worked in the area for many years and know the patients, their families and their villages intimately from providing counseling and health checks and giving out medication on a monthly basis. A few subjects had changed their first name, but it was not a problem for classification because most still lived in their original village. Missing of the specific date of last visited among the subjects, as a result could not use a better statistical model such as survival analysis including hazard model.

Prior to 2000, the majority of the Hill-tribe people did not have Thai identification cards, which is one reason for the poor attendance to clinics of this section of the study population during this period. Another important reason is that, for several years after HIV/AIDS diagnosis became recognized, the condition did not entitle the person to free medical care under the government’s universal medical care scheme. It is certain that many sufferers had never been diagnosed or that if they had been initially, they became lost to follow up due to financial constraints for treatment. The overall result of this study is that the recorded number of HIV patients who we were able to trace from records must have been lower than the true number in the beginning of the survey periods.

Other reasons that affected the number of recorded cases included social stigmatization in the early days, which deterred patients from seeking hospital care. Apidechkul in 2009 found that Akha youths, members of one of the Hill-tribes, preferred to travel to large cities to seek employment to support their families, and some contracted HIV/AIDS during their time away from home [11].

Previously available information from the Chiang Rai Public Health Office reported that between 1987 and 2009, the highest number of TB cases of HIV/AIDS in the province was recorded [12], 44.0 % of which were smear-positive pulmonary TB; among the TB infection patients, 24.0 % tested positive for HIV/AIDS. The Thai Public Health aimed at a TB treatment success rate of 85 %, but the actual success rate was 73.2 %, while it was only 64.7 % in those with HIV. Those who lived in remote areas had a greater default rate than those who lived near health centers [12], and default during TB treatment was a significant problem in HIV-infected patients [13]. Apidechkul in 2012 [14] reported that most of the TB infections in the Hill-tribe population, at 84.6 %, were pulmonary TB, and only 22.7 % were cured, while the HIV/AIDS prevalence among the Hill-tribe TB patients was 17.2 %. This report supports the results of the study that the Chain Rai province has a high prevalence of TB, particularly in HIV/AIDS patients.

Due to heavy stigmatization in the early period of HIV epidemic in Thailand, HIV with TB among the Hill-tribes would have been under-reported during the time. Amarita [15] reported that the confluence of the two epidemics rendered TB symbolic and symptomatic of HIV and enhanced the visibility of AIDS. Dual illness thus introduced a paradox to patients’ identity constructions and produced a unique, overlapping double stigma.

A study of Jimenez et al. (2006) found that men were more likely to get TB infection than women in southern Mexico [16]. However, the reports of Thorson et al. (2001), Young et al. (2009) and Holmes et al. (1998) found that women and girls were more likely to get TB and more serious outcomes because of delay in diagnosis [17–19].

Vermund et al. [20] reported that Southeast Asia faced the problem of TB-HIV/AIDS co-infection, particularly among MSM and IDU. However, in this study, the author found that the main route of transmission was sexual intercourse. Julien Zwang et al. [21] reported that the factors supporting the TB epidemic in Southeast Asia were poverty, living in crowded areas, and the quality and quantity of health care services and educational systems.

The number of TB reported cases increased steadily from 1990 to 2010, possibly due to inaccessibility of health care service and lack of awareness and technologies for diagnosis, and the policy of the Thai government of identifying new TB patients based on WHO guidelines [22]. The study also showed that youth (≤15 years old) had a smaller proportion of TB disease compared to those who ≥ 16. This might reflect the limited diagnostic options and that the numbers of HIV/AIDS patients in this group was less than in the others.

Kingkaew et al. [23] found that those with HIV/AIDS, especially males, were at greater risk for extrapulmonary TB; also in HIV patients, TB meningitis and low CD4 levels were risk factors for death. This result is inconsistent with our study, which found that the group without a diagnosis of TB had a greater chance of death compared to those with TB diagnosis. This paradox is most likely explained by the limited access to treatment of a marginalized population due to financial, social and/or legal problems, especially during the early period of HIV/AIDS. Mo et al. [24] also reported that patients with multiple infections with HIV, HBV, and HCV were at risk of developing abnormal LFTs and death during TB treatment. Unfortunately, the documentation in our study was not adequate for analyzing such associations.

The study also found that the Karen and Kmong tribes had a greater chance of death than the Yao. This may be due to their socio-economic status, which limits access to health care and causes them to be vulnerable to disease infections [25–27]. Karen and Kmong also widely use traditional medicine [28].

Lawn et al. [29] reported that the death rate of TB and HIV/AIDS co-infection dramatically decreased after scaling up HIV/AIDS testing among the TB-positive and improved the survival of those with HIV-associated TB, reducing mortality rates by 64 % to 95 %. This result agrees with our study, which found that patients receiving ARV had a greater chance of surviving. Arbor et al. [30] also reported that receiving ARV and co-trimoxazol and having CD4 greater than 50 were supportive factors for survival among HIV/AIDS patients. However, Boettigeretal et al. [31] reported that not everyone having ARV could control the viral load, as those patients who had a history of opportunistic infection tended to develop virological failure. A meta-analysis conducted by Jiang et al. [32] found that ARV could be associated with adverse events and drug interaction with antiTBs. However, Rangaka et al. [33] suggested that isoniazid preventive therapy should be recommended to all patients receiving antiretroviral therapy in moderate- or high-incidence areas. Mfinanga et al. [34] suggested that ART could be delayed until after the completion of six months of tuberculosis treatment for HIV-positive patients with tuberculosis who have CD4 cell counts greater than 220 cells per μL. Harries et al. [35] supported the early diagnosis and treatment of HIV infection in affected communities and proposed that the urgent assessment of frequent testing for HIV and early start of antiretroviral treatment (ART) should result in short-term and long-term declines in tuberculosis incidence through individual immune reconstitution and reduced HIV transmission. Yang et al. [36] reported that having started HAART during TB treatment was significantly associated with better survival. These regimens should be considered by the Thai public health services for implementation to improve the survival of HIV-TB co-infection in the Hill-tribe people.

Grenfell et al. [37] reported that the latent TB infection prevalence was high and that the active disease was more common among HIV-positive people with IDU. However, our study found only a small proportion of people with IDU and HIV/AIDS had TB infection among the Hill-tribes. The IDU is not a common route of choice among Hill-tribe drug abusers who prefer oral methamphetamine tablets [14].

We also found that being HIV and AIDS stages had a greater chance for death than those an asymptomatic stage. This is the natural history of HIV/AIDS disease in human; those at the HIV and AIDS stages have a greater chance of death than those at the early infection stage [38–40].

However, early in the HIV/AIDS epidemic in Thailand, people who knew their HIV status very often decided to commit suicide. Therefore, HIV positive and AIDS stages had a greater proportion of death than the asymptomatic stage. This is consistent with a report by Lotrakul [41] that the major cause of death among the HIV/AIDS patients in Northern Thailand was suicide. Porapakkham et al. [42], Guadamuz et al. [43], Schlebusch et al. [44], Bundhamcharoen et al. [45], and Chutinantakul et al. [46] also reported that suicide was most prevalent in the Upper Northern Thailand region, where HIV infection was related to the high prevalence of suicide and was the major cause of premature death.

The Hill-tribes of Thailand are minority groups with specific needs due to their language, cultural and social differences. Public health interventions to reduce the number of new TB and HIV/AIDS cases must take such needs into consideration in the planning and delivery of services, especially in health education. Thomas et al. [47] reported that using specific community-based social and behavioral counseling, as well as voluntary testing, could reduce the HIV incidence in Thailand and Africa. Thato et el. [48] also reported that a brief Peer-Led HIV Prevention Program significantly increased knowledge of preventive behaviors and motivated participants to have a better attitude toward preventive behaviors, better subjective norms, and greater intention to practice preventive behavior among school children in Bangkok, Thailand. Rojanawiwat et al. [49] reported that the National Access to Antiretroviral Program reduced the number of opportunistic infections, especially PCP and TB, in Thai people living with HIV/AIDS.

## Conclusion

In conclusion, the present study indicates that being Akha and female and lacking access to ARV were the major causes of TB infection among the HIV/AIDS Hill-tribe population. Our study suggests an urgent need to consistently implement TB and HIV control programs for the Thai Hill-tribe.

## Abbreviations

AIDS: acquired immune deficiency syndrome
ART: antiretroviral therapy
ARV: antiretroviral
CBC: complete blood count
CD4: cluster of differentiation 4
CI: confident interval
CVI: content validity index
HAART: highly active antiretroviral therapy
HBV: hepatitis B virus
HCV: hepatitis C virus
HIV: human immune deficiency virus
ID: identification number
IDU: injecting drug users
MDG: millennium development goals
OI: opportunistic infections
OPD: outpatient department
PCP: pneumocystis pneumonia
TB: tuberculosis

## References

[1] United Nations (2014). The Millennium Development Goals Report 2014.

[2] World Health Organization (WHO). Fact sheet: HIV/AIDS November 2014. 2014. http://www.who.int/mediacentre/factsheets/fs360/en/.

[3] World Health Organization (WHO). Fact sheet: Tuberculosis October 2014. 2014. http://www.who.int/mediacentre/factsheets/fs104/en/

[4] United Nations Thailand. Thailand population 2014. 2014. http://un.or.th/thailand/population.html

[5] UNAIDS. HIV/AIDS estimation 2012. 2012. http://www.unaids.org/en/regionscountries/countries/thailand/

[6] Bureau of Epidemiology, Ministry of Pubic Health. HIV/AIDS situation in Thailand 2014. 2014. http://www.boe.moph.go.th/files/report/20141128_61345755.pdf

[7] World Health Organization (WHO). Fact sheet TB-HIV: HIV-associated Tuberculosis. 2014. http://www.who.int/tb/challenges/hiv/tbhiv_factsheet_2014.pdf

[8] World Health Organization (WHO). Tuberculosis in Thailand. Bangkok: WHO Country office for Thailand. 2014. http://www.searo.who.int/thailand/areas/tuberculosis/en/

[9] Princess Maha Chakri Siridhorn Anthropology center. Hill tribe. 2014. http://www.sac.or.th/main/index.php

[10] The hill tribe welfare and development center, Chiang Rai province. Hill tribe population (2011). The hill tribe welfare and development center.

[11] Tawatchai K, Sukhum J, Sutthi J, Jaranit K (2007). Sexual behavior and HIV infection among pregnant hill tribe women in northern Thailand. Southeast Asian J Trop Med Public Health.

[12] Chiang Rai Public Health Office (2010). TB and HIV/AIDS Report 2010. Chiang Rai: Chiang Rai Public Health Office. Chiang Rai page.

[13] Kittikraisak W, Burapat C, Kaewsa-ard S, Wattanaamornkiet W, Sirinak C, Sattayawuthipong W (2009). Factors associated with tuberculosis treatment default among HIV-infected tuberculosis patients in Thailand. Trans R Soc Trop Med Hyg.

[14] Apidechkul T (2012). TB/HIV among the hill tribe marginalized vulnerable population, Thailand. Int J Infect Dis.

[15] Daftary A (2012). HIV and Tuberculosis: The construction and management of double stigma. Soc Sci Med.

[16] ME J –C, Garcia-Garcia L, DeRiemer K, Ferreyra-Rayes L, Bobadilla-del-Valle M, Cano-Arellano B (2006). Gender differentials of pulmonary tuberculosis transmission and reactivation in an endemic area. Thorax.

[17] Thorson A, Diwan VK (2001). Gender inequalities in tuberculosis: aspects of infection, notification rates, and compliance. Curr Opin Pulm Med.

[18] Fiona Y, Julia AC, Lucy KJ, Nigel CU (2009). A review of co-morbidity between infectious and chronic disease in Sub-Saharan Africa: TB and diabetes mellitus, HIV and metabolic syndrome, and the impact of globalization. Glob Health.

[19] Holmes CB, Hausler H, Nunn P (1998). A review of sex difference in the epidemiology of tuberculosis. Int J Tuberc Lung Dis.

[20] Sten V, Naoki Y (2007). Co-infection with human immunodeficiency virus and tuberculosis in Asia. Tuberculosis.

[21] Zwang J, Garenne M, Kahn K, Collinson M, Tollman ST (2007). Trends in mortality from pulmonary tuberculosis and HIV/AIDS co-infection in rural South Africa (Agincourt). Trans Royal Society Trop Med Hyg.

[22] World Health Organization (WHO). Guidelines for intesified tuberculosis case-finding and isoniazid preventive therapy for people living with HIV in resources-constrained settings. Jeneva: WHO. 2010. http://apps.who.int/iris/bitstream/10665/44472/1/9789241500708_eng.pdf

[23] Kingkaew N, Sangtong B, Amnuaiphon W, Jongpaibulpatana J, Mankatittham W, Akksilp S (2009). HIV-associated extrapulmonary tuberculosis in Thailand: epidemiology and factors for death. Int J Infect Dis.

[24] Mo P, Zhu Q, Teter C, Yang R, Deng L, Yan Y (2014). Prevalence, drug-induced hepatotoxicity, and mortality among patients multi-infected with HIV, tuberculosis, and hepatitis virus. Int J Infect Dis.

[25] Apidechkul T (2015). Prevalence and risk factors of intestinal parasitic infections among hill tribe schoolchildren, northern Thailand. Asian Pacific Tropical Disease.

[26] Nacher M, Singhasivanon P, Vannaphan S, Treeprasertsuk S, Phanumaphorn M, Traore B (2001). Socio-economic and environmental protective/risk factors for severe malaria in Thailand. Acta Trop.

[27] Kunstadter P, Kunstater SL, Podhisita C, Leepreecha P (1993). Demographic variables in fetal and child mortality: Hmong in Thailand. Soc Sci Med.

[28] Tangjitman K, Wongsawad C, Winijchaiyanan P, Sukkho T, Kamwong K (2013). Traditional knowledge on medicinal plant of the Karen in northern Thailand: A comparative study. J Ethnopharmacol.

[29] Lawn SD, Kranzer K, Wood R (2009). Antiretroviral Therapy or Control of the HIV-associated Tuberculosis epidemic in resource-limited settings. Clin Chest Med.

[30] Agbor AA, Joel JRB, Billong SC, Tejokem MC, Ekaila GL, Plottel CS (2014). Factors Associated with Death during Tuberculosis Treatment of Patients Co-Infected with HIV at the Yaounde’ Central Hospital, Cameroon: An 8-Year Hospital-Based Retrospective Cohort Study (2006–2013). PLoS One.

[31] Boettiger DC, Kerr S, Ditangco R, Merati TP, Thanh-Pham TT, Chaiwath R (2014). Trends in first-line antiretroviral threapy in Asia: Results from the TREAT Asia HIV observatinal database. PLoS One.

[32] Jiang J, Yang X, Ye L, Zhou B, Ning C (2014). Pre-exposure prophylaxis for the prevention of HIV infection in high risk population: a meta analysis of randomized controlled trial. PLoS One.

[33] Molebogeng XR, Wilkinson RJ, Boulle A, Glynn JR, Fielding K, Cutsem GV (2014). Isoniazid antiretroviral therapy to prevent tuberculosis a randomized double-blind, placebo-controlled trail. Lancet.

[34] Mfinanga SG, Kirenga BJ, Chanda DM, Mutayoba B, Mthiyane T, Yimer G (2014). Early versus delayed initiation of highly active antiretroviral therapy for HIV-positive adults with newly diagnosed pulmonary tuberculosis (TB-HAART): a prospective, international, randomized, placebo-controlled trail. Lancet Infect Dis.

[35] Harries AD, Zachariah R, Corbett EL (2010). The HIV-associated tuberculosis epidemic–when we will act?. Lancet.

[36] Yang CH, Chen KJ, Tsai JJ, Lin YH, Cheng SH, Wang KF (2014). The impact of HAART initiation timing on HIV-TB co-infected patients, a retrospective cohort study. BMC Infect Dis.

[37] Grenfell P, Leite RB, Garfein R, Lussigny SD, Platt L, Rhodes T (2013). Tuberculosis, injecting drug use and integrated HIV-TB care: A review of the literature. Drug Alcohol Depend.

[38] Jonsthapongpanth A, Bagchi-Sen S (2010). Spatial and sex differences in AIDS mortality in Chiang Rai. Thailand Health & Place.

[39] Rapose A, East J, Sova M, O’Brien WA (2008). AIDS: Disease manesfation. Encyclopedia of virology (3rd edition).

[40] Rotheram-Borus MJ, Swendeman D, Amani B, Applegate E, Milburn NG, Arnold EM (2011). AIDS. Encyclopedia of adolescence.

[41] Lotrakul M (2006). Suicide in Thailand during the period 1998–2003. Psychiatry Clin Neurosci.

[42] Porapakkham Y, Rao C, Pattaraarchachai J, Polprasert W, Vos T, Adair T (2010). Estimated causes of death in Thailand, 2005: implications for health policy. Population Health Metrcis.

[43] Guadamuz TE, McCarthy K, Winmonsate W, Thienkrua W, Varangrat A, Chaikummao S (2014). Psychosocial health conditions and HIV prevalence and incidence in a cohort of men who have sex men in Bangkok, Thailand: Evidence of a syndemic effect. AIDS Behav.

[44] Schlebusch L, Govender RD (2012). Age, gender and sucicide ideation following voluntary HIV counseling and testing. Int J Environ Res Public Health.

[45] Bundhamcharoen K, Odton P, Phulkerd S, Tangcharoensathien V (2011). Burden of disease in Thailand: changes in health gab between 1999 and 2004. BMC Public Health.

[46] Chutinatakul A, Tongkumchum P, Budhamcharoen K, Chongsuvivatwong V (2014). Correcting and estimating HIV moratality in Thailand based on 2005 verbal autopsy data focusing on demographic factoirs, 1996–2009. Popul Health Metrics.

[47] Coates TJ, Kulich M, Calentano DD, Zelaya CE, Chariyalertsak S, Chingono A (2014). Effect of community-based voluntary counseling and testing on HIV incidence and social and behavioral outcomes: a cluster-ransomed trail. The Lancet Global Health.

[48] Thato R, Penrose J (2013). A brief, peer-led HIV prevention program for college- students in Bangkok, Thailand. J Pediatr Adolesc Gynecol.

[49] Rojanawiwat A, Tsuchiya N, Pathipvanich P (2011). Impact of the national access to antiviral program on the incidence of opportunistic infections in Thailand. International Health.

